# Quality of Sexual Life after Native Tissue versus Polypropylene Mesh Augmented Pelvic Floor Reconstructive Surgery

**DOI:** 10.3390/jcm10214807

**Published:** 2021-10-20

**Authors:** Aleksandra Kamińska, Katarzyna Skorupska, Agnieszka Kubik-Komar, Konrad Futyma, Joanna Filipczak, Tomasz Rechberger

**Affiliations:** 1 2nd Department of Gynecology, Medical University of Lublin, 20-950 Lublin, Poland; kasiaperzylo@hotmail.com (K.S.); futymakonrad@mp.pl (K.F.); rechbergt@yahoo.com (T.R.); 2 Department of Applied Mathematics and Computer Science, University of Life Science, 20-033 Lublin, Poland; agnieszka.kubik@up.lublin.pl; 3 Medical University of Lublin, 20-059 Lublin, Poland; joannafilipczak15@onet.pl

**Keywords:** PISQ-12, FSFI, pelvic organ prolapsed, transvaginal mesh, vaginal native tissue repair

## Abstract

There are still controversies around reconstructive surgeries used in POP treatment. The aim of this study was to compare the QoSL after VNTR vs. TVM surgery due to POP via the use of PISQ-12 and FSFI questionnaires. The study included a group of 121 sexually active patients qualified for reconstructive surgery due to symptomatic POP, and 50 control. The average results of PISQ-12 before and after surgery were compared using the *t*-test. The significance of the mean differences in demographic groups was measured using the *t*-test for independent samples and one-way ANOVA. The results in the demographic groups were compared using the Mann–Whitney U test and the Kruskal–Wallis test. Fifty-eight women had VNTR, while 63 had TVM. Results of PISQ-12 revealed significant improvement in the sexual life after reconstructive surgery (27.24 vs. 32.43; *p* < 0.001, *t* = 8.48) both after VNTR and TVM. There were no significant differences in the assessment of the QoSL according to PISQ-12 and FSFI results between both analyzed groups of patients (PISQ-12: VNTR vs. TVM; *t*-test *p* = 0.19 and FSFI: VNTR vs. TVM; Mann–Whitney U test *p* = 0.54). VNTR is the treatment of choice in the case of uncomplicated primary POP.

## 1. Introduction

Pelvic organ prolapse (POP) is defined as pelvic organ descent caused by biochemical disorders in the ligaments, fascia, and muscles of the pelvic floor that leads to the displacement of the vagina or cervix towards or outside the hymen, and even to total vaginal or uterine prolapse [1]. The real prevalence rate of POP is difficult to estimate as this depends to a large extent on the characteristics of the examined population, the research methods and the definition. It can, however, range from 3% to even 50%, whereby POP defined according to symptoms occurs in about 3%–6% of all women, compared with 41%–50% of all diagnoses made during gynecological examination [2].

Controversies around reconstructive surgeries used in POP treatment, regarding both procedures performed with the use of native tissues (vaginal native tissue repair, VNTR) and synthetic materials are widely presented in the literature [3,4,5,6,7,8]. Despite this fact, it is necessary to conduct further research on the impact of the defect on the quality of female sexual life and functional disorders in patients undergoing POP surgical repair.

Assessment of the patient’s subjective perception of health and the quality of life (QoL) is necessary to correctly determine the needs and expectations of patients and to properly plan and choose treatment methods tailored to a specific person. Still, medical history, together with physical examination, including the measurement of the length and width of the vagina, when used to assess sexual function, proved to be an insufficient predictive tool. Therefore, in addition to physical examination, the functional aspects of POP should be ascertained using validated questionnaires to create a complete plan of treatment. 

The popularity of validated questionnaires assessing the impact of pelvic floor disorders (PFD) on women’s sexual health has been increasing for about 20 years [9]. Research indicates that the impact of PFD on female sexual health can be established by using validated questionnaires in the preoperative period [10]. Although these tools, both disease-specific and general QoL appraisal, have been translated into different languages, certain restrictions connected to cultural and religious variability must always be taken into account [11].

Questionnaires filled out by patients are a discreet, reproducible method of assessing women’s sexual life. Of course, it should be remembered that sexuality is multidimensional, therefore, simply obtaining a result in a given questionnaire cannot capture the complexity of the problem.

General questionnaires, such as the female sexual function index (FSFI) are designed to assess sexual function in the general population and may, therefore, not be accurate enough when detecting differences related to ailments in a group of people with specific diseases [12].

Questionnaires designed for specific medical conditions, such as pelvic organ prolapse or urinary incontinence sexual questionnaire (PISQ-12), are necessary to compare sexual functions within the population of patients with urinary incontinence (UI) or POP. They are also indispensable tools for assessing the impact of treatment on women sexual life [13].

The aim of this study was to compare the quality of sexual life after native tissue versus polypropylene mesh reconstructive surgery due to POP.

## 2. Materials and Methods

### 2.1. Sample Size

Subject-to-item ratio of at least 5:1 has been recommended during performing psychometrics and scale evaluation [14,15]. In our work, the used PISQ-12 version contained 12 items. Therefore, a sample size of at least 60 was required to fulfill the above criteria and assess the correlation of the scales with the FSFI and objective measures of different PISQ-12s. However, taking into consideration the variety of statistical tests performed in this study, we enlarged the sample size to provide approximately 0.8 power of each test at the assumed effect size.

### 2.2. Study Population

The study included a group of 121 sexually active patients who had undergone surgery due to symptomatic POP at the tertiary gynecological department between October 2015 and February 2018. As control, 50 women without POP complaints were also enrolled. Patients underwent the full clinical assessment before the procedure and 6 months after surgery. Urogynecological examination for both groups were performed according to the International Continence Society (ICS) standards [16,17]. This included gynecological and ultrasound examination with cough test without and with reposition of prolapse and assessment of POP degree according to the pelvic organ prolapse quantification scale (POP-Q) [18]. Moreover, patients from the study group were asked to complete questionnaires twice, before and after surgery, the patients from the control group completed the forms only once. All patients were able to understand and complete the questionnaires themselves. In addition, all study participants were informed about the risks, benefits, and alternative treatment options, and signed an informed consent.

### 2.3. Surgical Techniques

The surgical technique in case of anterior defect ≥III POP-Q stage included an anterior median longitudinal colpotomy in order to reach the pubocervical fascia after performing a vaginal submucosal infiltration with diluted epinephrine solution. The anterior repair was performed by placing two layers of 2–0 synthetic absorbable sutures at the pubocervical fascia. The excess of the vaginal wall was removed and the vaginal mucosa was closed with continuous absorbable suture. In case of posterior defect ≥II POP-Q stages, a vertical incision in the posterior vaginal mucosa was made and the rectovaginal fascia was identified and reconnected to the uterosacral ligaments at the top of the vagina and finally sutured to the iliococcygeus fascia and muscle inferiorly to the ischial spines. The rectovaginal fascia was repaired with single absorbable sutures. Finally, reconstruction of the perineal body was performed. Vaginal skin closure was performed with delayed absorbable sutures [19].

In case of implantation of transvaginal polypropylene meshes (TVM), surgery was performed depending on the type of disorder defined in the POP-Q scale.

In case of TVM anterior, the anterior arms were inserted through the fascia and muscular structures 1/3 of the upper obturator foramen (mainly the fascia of the external obturator muscle) and the posterior arms were inserted through the arcus tendinous fasciae pelvis (ATFP) approximately 1 cm proximally from the ischial spine and brought out by obturator foramens onto the skin.

In the case of TVM posterior, mesh arms were inserted through the sacrospinous ligaments approximately 1 cm medially from the ischial spine, guided through the ischioanal fossa on both side of the rectum and brought on the skin approximately 3 cm laterally and 3 cm below the external anal sphincter. Mesh was sutured with a non-absorbable suture to the cervix in order to prevent from uterus prolapse recurrence. Vaginal skin closure was performed with delayed absorbable sutures.

TVM modified procedure, consisting in the implantation of a modified 4-arm TVM mesh used in the case of advanced lowering of the vaginal apex in patients after hysterectomy. At the start of the passage, the trocar will perforate the obturator externus muscle and then the obturator membrane. The device should then be advanced medially through the obturator membrane and pass through the obturator internus muscle approximately 1 cm from the proximal end of the ATFP. At that point, the anterior arm of the mesh is retrieved and the middle upper part of the mesh is sutured to the pubocervical fascia. The posterior arms of the mesh are positioned in the ischioanal fossas, inferior to levator ani muscle, and secured by passage of both arms through the sacrospinous ligaments and coccygeus muscles [20]. 

The study protocol was approved by the Local Bioethics Committee (KE-0254/42/2016).

### 2.4. Questionnaires

The PISQ-12 is a shortened version of the PISQ-31 questionnaire presented in 2001 by Rebecca Rogers and subjected to validation into Polish in 2017 [13]. PISQ is the only questionnaire created to assess the sexual function of patients with UI or POP. The reliability of PISQ-12 in the population of women with PFD was classified to grade A according to ICS [21]. This questionnaire is used to assess sexual function in heterosexual patients with diagnosed POP or UI who were sexually active over the last 6 months. It should not be used in patients who have no partner or are sexually inactive. According to the authors, it is recommended to interpret the results of PISQ-12 as the sum of sexual function. The maximum score is 48 points. The higher the score, the better the quality of sex life [1].

FSFI is a Polish-validated questionnaire used to appraise sexual functioning in the general population. It was developed to assess sexual desire, orgasm, sexual satisfaction, and pain in clinical trials. It is not recommended to use the questionnaire as a diagnostic tool. Moreover, it should not be used as a substitute for a full sexual history in clinical judgment, as it does not measure sexual experience, knowledge, attitude, or interpersonal relationships.

The relationship between the results obtained and the assessment of sexual functions is directly proportional, i.e., the higher the number of points, the better sexual functioning, in the domain of pain, the higher the score, the less pain. Scores range from 2 to 36 points [22]. Clinically significant sexual dysfunctions within the Polish population are diagnosed with a score of 27.50 or less. Research shows that the Polish version of the FSFI questionnaire is a reliable tool with good psychometric values [12]. In contrast to the PISQ-12 questionnaire described above, the FSFI has response options dedicated to sexually inactive patients, but it cannot be used to assess the effect of POP on sexual function.

### 2.5. Statistical Analysis

The data analysis included the determination of the basic characteristics of the distribution PISQ-12 and FSFI results, as well as checking, on the basis of statistical tests, whether these distributions differ significantly before (T1) and after (T2) surgery and in demographic and other studied groups.

The normal distribution was tested using the Shapiro–Wilk test. On the basis of its results parametric vs. nonparametric tests were chosen for further analysis. 

The *t*-test was used to assess the homogeneity of the groups in terms of age and BMI.

The average results of PISQ-12 before and after surgery were compared using the *t*-test for dependent variables.

Nonparametric tests were used for FSFI results. The Wilcoxon pair test allowed to compare patient responses before and after surgery. The differences in the assessment of the quality of sexual life between patients qualified for VNTR and TVM surgery were compared using the Mann–Whitney U test. The results of the FSFI questionnaire between patients undergoing different types of surgery were compared using the Kruskal–Wallis test.

The convergence of results from both questionnaires was assessed by determining the value of the Spearman correlation coefficient.

## 3. Results

Patient demographical and clinical characteristics are presented in Table 1.

The majority of patients, 68% (*n* = 82) in the study group were after menopause, while the remaining 32% (*n* = 39) were before menopause. In the control group, regular monthly bleeding was declared by 46% of all women surveyed, the remaining 54% were after menopause.

Among the patients qualified for surgical treatment, 58 women had VNTR, whereas 63 had transvaginal mesh surgery (TVM).

The groups were demographically homogeneous. 

Among the analyzed demographic groups there was significant statistical difference between mean age in VNTR and TVM patients with *p* = 0.03, which is not so distant from the significance level α = 0.05. This indicates a discreet difference between the studied groups (Table 1). 

In the VNTR group, the most frequently performed procedure was anterior and posterior kolporraphy (*n* = 39), followed by posterior kolporraphy alone (*n* = 8), Forthergill operation (*n* = 7) and anterior vaginal wall kolporraphy (*n* = 4).

In the TVM group, 30 patients had polypropylene mesh placed beneath the pubocervical or rectovaginal fascia, 20 patients underwent only anterior and 3 only posterior mesh placement. In 10 patients, four arm mesh was used due to advanced vaginal cuff prolapse after hysterectomy (TVM modified anterior arms were introduced through the obturator foramen, whereas the posterior arms were repositioned via the ischiorectal fossa and uterosacral ligament). This was followed by the reconstruction of the perineal body.

Results of PISQ-12 questionnaire application within the entire analyzed population revealed that significant improvement occurred in their sexual lives after reconstructive surgery both after VNTR and TVM (Table 2).

The FSFI questionnaire results also showed statistically significant improvement in all analyzed domains, as well as in the overall results (Table 3).

There were no significant differences in the assessment of the sexual QoL according to PISQ-12 questionnaire between both analyzed groups of patients (VNTR vs. TVM; *t* = 1.29, *p* = 0.2).

What is more, no significant differences existed in the assessment of the sexual QoL in FSFI between the patient groups after VNTR and TVM (VNTR vs. TVM Z = 0.58, *p* = 0.95).

**Figure 1:**
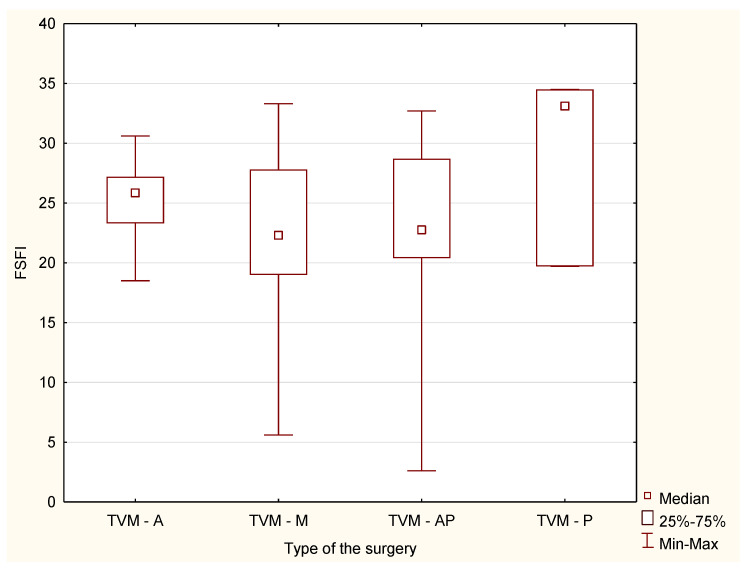
Median and range of FSFI results after TVM surgery depending on the place of mesh placement. TVM-A—TVM anterior; TVM-P—TVM posterior; TVM-AP—TVM anterior and posterior; TVM-M—modified transvaginal mesh.

**Figure 2:**
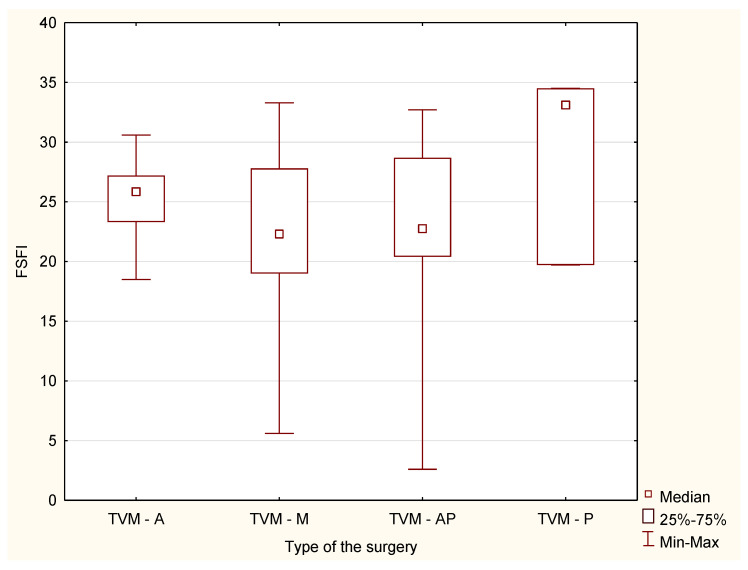
Median and range of FSFI results after VNTR depending on the type of surgery. A-C—anterior colporraphy; P-C—posterior colporraphy; AP-C—anterior and posterior colporraphy; F-O—Fothergill’s operation.

There were also no statistically significant differences in the responses between the study groups in the case of division into specific surgical procedures in the field of mesh implantation and colporraphy (H = 0.95, *p* = 0.81 and H = 0.13, *p* = 0, 98, respectively) (Figure 1 and Figure 2).

The correlation between PISQ-12 and FSFI before qualification for surgery (T1) was 0.63, and after surgery (T2)—0.76. The results of the correlation, taking into account the type of surgery performed, are presented in Table 4.

In the analysis of the mutual correlation between the PISQ-12 and FSFI questionnaires, the obtained results indicated a greater compatibility of the assessment of the quality of sexual life using both questionnaires in the group of patients after surgery, both in the case of group analysis regardless of the type of surgery performed, and when specifying the types of operations. This fact may explain the subjective feeling of improvement in the self-esteem of patients that was conditioned by the anatomical defect.

## 4. Discussion

It is estimated that approximately 11% of all women after vaginal deliveries will be operated on due to PFD [23]. Until now, most of the researches focused on the anatomical non-functional results or on the occurrence of dyspareunia, but not on patient sexuality. An analysis of the available literature suggests a statistically significant improvement in dyspareunia, as well as no impact or improvement in the quality of sex life after surgery. Thus, physical changes after surgery may not take into account problems that cause sexual dysfunction before surgery, and may not bring the desired results [24,25].

Surgical procedures used in reconstructive surgery of the POP have developed over the recent years, and there is a wide range of treatment options. This allows adapting the method to the defect, as well as to patient expectations. Due to warnings issued by the US Food and Drug Administration (FDA) initiated in 2008 and numerous collective legal proceedings regarding the implantation of synthetic meshes, many doubts and fears have arisen, above all regarding potential complications typical for this type of surgery and persisting even after removal of the material. In 2010, with a 2014 update, Health Canada confirmed the FDA’s position, but to a less radical extent, stating that although most patients operated with TVM had good results, there was an increased risk of complications compared to traditional repair procedures.

Because of the lower rate of recurrent prolapse and less risk of dyspareunia compared with vaginal approach, the abdominal promontofixation is the criterion standard surgical procedure for correction of the uterovaginal prolapse. The laparoscopic type of the surgery is associated with quicker postoperative recovery and shorter hospital stay, decreased pain and blood loss. However, laparoscopic promontofixation with mesh used to suspend the central compartment to the anterior longitudinal ligament of the sacrum involves the risks of complications associated with the mesh use. Seracchioli et al. performed mesh-less supracervical laparoscopic hysterectomy with suspension of the cervix to the anterior longitudinal ligament of the sacral promontory through a continuous suture with plication and shortening of the right uterosacral ligament. They evaluated surgical and clinical outcomes of the procedure with using validated questionnaires. At 12 months of follow-up, they found that overall FSFI scores improved significantly (*p* = 0.002) which confirms the improvement in the quality of sexual life of the patients after surgery. Before surgery, 16 (34.8%) of patients had a sexual life-related distress (FSFI score 26.5 or lower), after surgery only 1 patient had persistent sexual disfunction. What is more, no women complained from „de novo” dyspareunia during follow-up [26]. 

There is a new technique for POP surgery described with comparable outcomes to those of laparoscopic sacral colpocervicopexy. In the pectopexy procedure, lateral parts of the iliopectineal ligament are used for cuff or cervix suspension and the laparoscopic pectopexy is a good alternative to sacral colpopexy as a gold standard for apical prolapse. Tahaoglu et al. examined the early outcomes of this procedure and evaluated its effects on female sexual function and QoL with using FSFI and prolapse quality of life (P-QOL) questionnaires. All FSFI and P-QOL scores improved significantly (*p* < 0.05) 6 months after surgery, but mean total FSFI score postoperatively (23.63 ± 4.67) still showed sexual dysfunction (<26.55). Sarlos et al. showed significant improvement after laparoscopic sacrocolpopexy with negligible de novo dyspareunia. Laparoscopic pectopexy shows promising results for apical defect as an alternative surgery to sacrohysteropexy [27,28,29]. 

An interesting conclusion explaining the discrepancies in the assessment of the QoL after procedures using polypropylene mesh is the study of Kelly et al. which clearly shows the huge impact of the operator’s skills and experience on the potential success of surgery [5]. The guidelines of the American Urogynecologic Society (AUGS) emphasize the importance of interest in the patient after surgery and in obtaining fully informed consent for surgery [30].

Postoperative results cannot be accurately predicted, as evidenced by the occurrence of dyspareunia or de novo pain in the later postoperative period, and taking into account existing sexual dysfunctions is necessary when discussing with patients the type of surgery and the risks connecting with operation [25,30].

According to the available literature, the most frequently used tool for assessing the QoL after reconstructive surgery performed due to POP is the PISQ-12 questionnaire. The FSFI questionnaire, which aims to measure physiological and affective aspects of stimulation and sexual activity, although it is not a POP-specific test, is also often used for this type of analysis. In fact, in the study of Aube and Tu [4], FSFI was shown to have very good correlation with PISQ-12 results. 

The use of transvaginal meshes in operations due to POP still remains controversial, while the available literature emphasizes the fact that this type of surgery is more effective compared to VNTR surgery, especially in the context of anatomical recurrence of a POP [31]. In our study, we compared the sexual life satisfaction of patients after VNTR versus TVM.

Preoperative counselling includes discussion about different approaches including VNTR, mesh laparoscopic and abdominal techniques and transvaginal mesh implantation. After explanation and analyze potential benefits and the risk of serious complication, patients are asked to signed the informed consent to the procedure.

Based on the analysis of the results obtained from the PISQ-12 and FSFI questionnaires, we did not find statistically significant differences in the quality of sex life after VNTR and TVM (31.64 ± 6.7 and 33.15 ± 6.22, respectively; *p* = 0.19 for PISQ-12 and 23.42 ± 7.64 and 24.20 ± 6.29; *p* = 0.55 for FSFI). Further analysis, including specific types of procedures divided into compartments, also showed no differences in the aspect of sexuality, despite the treatment method used.

The conclusions of the presented study confirmed the results obtained by Sao-Cun Liao et al. involving 2976 patients (1488 after surgery using TVM and 1488 after VNTR). In their study, there was no significant difference in postoperative pain after TVM compared to classical surgery and no statistically significant difference in de novo dyspareunia between both study groups. Our study revealed similar postoperative results for both sexual function and the occurrence of dyspareunia regardless of the type of surgery [32].

Both in the present study and in the available literature, the negative impact of symptomatic POP on QoL of women was clearly demonstrated. Therefore, the potential risk of recurrence of the symptoms despite the treatment used is not insignificant. Although the lifetime risk of surgery for POP is 11.1%, there is a high probability of surgery failure. It is estimated that 3 out of 10 women operated with classical methods require reoperation, and 60% of all relapses are observed in the same compartment [23,33]. The degree of advancement of vaginal wall descent >2 according to the POP-Q scale, previous reconstruction operations and a defect involving several compartments are the most important risk factors for recurrence.

In the USA, the most common type of surgery for patients with POP in 2003, was anterior and posterior vaginal colporraphy, which was performed in 35.2% of all women with this condition. Within 4 years of primary surgery, the recurrence rate after traditional surgery was 29.2% [34].

In most prospective randomized studies, the benefits of using meshes to treat anterior vaginal defect have been demonstrated. The assessment of the effects of reconstruction 12 months after the surgery showed that a statistically significant improvement was obtained when prostheses were used rather than classical anterior colporraphy (89% vs. 64%, *p* = 0.0006). Additionally, relapses in both functional and anatomical aspects were less common in patients after mesh implantation (31.3% vs. 52.2%, *p* = 0.007). Indeed, 2 out of 72 patients after traditional colporraphy had to be reoperated, compared to 1 patient qualified for re-surgery and cutting of the mesh arms due to dyspareunia 8 months after implantation of TVM. The percentage of vaginal wall perforation with synthetic material exposure was 9.5%, while de novo dyspareunia (1 in 14 patients after VNTR, 3 in 13 after implantation of synthetic materials) was found in both groups. According to de Tayrac et al. [6], the improvement of QoL assessed on the basis of questionnaires was in both groups of patients to be without a significant difference between them. Still, in the study conducted by Altman et al., statistically significantly better postoperative results after 12 months were found in patients operated with synthetic materials compared to VNTR (60.8% vs. 34.5%, *p* < 0.001) [7].

A summary of the results of treating anterior vaginal wall defect with a collagen coated mesh compared to the classical method shows that the use of meshes is associated with significantly better anatomical results, as indicated by a smaller number of women with grade 2 or higher anterior vaginal prolapse during a 12-month period observation. In contrast, in a group of patients after classical surgery, 48% had an anatomical defect in stage 2 or higher 3 months after surgery, reaching 60% after a year. In comparison, in a group after implantation of synthetic materials, these results were 4% and 14%, respectively. In this study, there were no differences in sexual function assessed on the basis of the PISQ-12 results between the examined groups. However, the study authors claim that the lack of differences might be due to the age of patients (the average age in the group qualified for classical surgery was 64.9 ± 6.4 years, and for surgery using synthetic materials 64.7 ± 6.6) and the associated lower sexual activity. Interestingly, despite observing a higher percentage of symptoms associated with POP after classical operations, these patients did not see the need for reoperation. This is in contrast to the reports of Barber et al. who put forward the thesis of defining postoperative success only when the symptoms of vaginal wall lowering are completely abolished [35,36].

In 2016, the largest, randomized, multicenter PROSPECT study was published, comparing the results of surgical treatment using synthetic mesh implantation or biological transplants and classic operations using patients’ own tissues. Analysis of the results showed a lower percentage of symptomatic POP and reoperation after using non-absorbable synthetic meshes than after VNTR, but no sufficiently reliable data were available on which treatments improved the QoL to a greater extent. There was also no significant difference in postoperative effects and results regarding the QoL between the examined groups after 1 year after surgery. However, serious doubts about the conclusions that can be drawn from the PROSPECT trial results have been indicated [37].

The PROSPECT study leads to the conclusion that the use of non-absorbable biological meshes or grafts in the treatment of PFD does not bring potentially greater benefits, and despite a similar percentage of postoperative complications, mesh implantation may generate unnecessary risk of reoperation due to exposure of material to the vagina in the first two years after surgery [8].

In the context of the ongoing debate on the deterioration of sexual function after reconstructive operations using synthetic materials, Morselli et al. assessed the quality of sex life in the study involving 155 women with POP disorders of less than grade 3 according to POP-Q scale qualified for TVM implantation. Based on the results obtained after completing the FSFI questionnaire 12 months after surgery, no adverse effect on sexual function was found. There was also evidence of dyspareunia in only one patient and statistically insignificant changes in points obtained in the pain domain (*p* = 0.124). The authors emphasize the importance of surgeon experience for the successful postoperative effect [38].

El Haddad et al. [39] in 2013, came to similar conclusions based on the analysis of the results of the PISQ-12 before and 6 months after surgery with the use of synthetic materials. The authors showed no deterioration of sexual function, a significant improvement in QoL and a low incidence of de novo dyspareunia (4% of all examined women) and a slight increase in the percentage of reported pain after treatment (29%), compared to the results from the first visit (25%) [39].

Due to the fact that the studies did not show significant differences in patient satisfaction, QoL nor anatomy of the genital organs after VNTR or mesh augmented surgery, using own tissues is the treatment of choice in the case of uncomplicated primary POP, while TVM is the treatment of choice in patients with relapse, but is also first-line treatment in the presence of evident risk factors for relapse [8]. This opinion is consistent with the thesis formulated by Scientific Committee on Emerging and Newly Identified Health Risks (SCENIHR) in 2015: “Based on available scientific evidence, due to the increased risk associated with the use of TVM in the treatment of POP, meshes should be used only when other surgical procedures fail”.

Regarding our study, its limitation was the single setting. The strength was the fact that this was a prospective study and the fact that the same team of experienced urogynecological surgeons performed all surgeries.

## 5. Conclusions

After the results analysis, the following conclusions were obtained: pelvic organ prolapse has a significant impact on the deterioration of the quality of sexual life of women. Pelvic reconstructive surgery regardless of its type improve the quality of sexual life of patients. Both after classic surgeries with the use of own tissues and synthetic materials, the quality of life of patients with pelvic organ prolapse is improved. There were no significant differences in improving the quality of life of patients between the group of women operated upon in a classic way and using synthetic materials.

It can be concluded that VNTR is the treatment of choice in the case of uncomplicated primary POP. 

## Figures and Tables

**Table 1:** Patient demographic and clinical characteristics.

	VNTR	TVM
Patients	*n* = 58	*n* = 63
BMI (Mean ±SD)	27.12 ± 4.2	26.06 ± 2.9
Age (Mean ±SD)	52.2 ± 8.6	55.6 ± 8.3
Menopausal status (pre- and post- menopause)	20 vs. 38	19 vs. 44

VNTR—vaginal native tissue surgery, TVM—transvaginal mesh, SD—standard deviation, *n*—number of patients.

**Table 2:** Mean results of the pelvic organ prolapse or urinary incontinence sexual questionnaire (PISQ-12) scoring before and 6 months after both types of reconstructive surgery.

PISQ-12	Before	After 6 Months	*p*
All			
Mean ± SD	27.24 ± 7.85	32.43 ± 6.48	<0.001
VNTR			
Mean ± SD	27.40 ± 7.32	31.64 ± 6.71	<0.001
Synthetic material			
Mean ± SD	27.09 ± 8.37	33.16 ± 6.23	<0.001

SD—standard deviation, VNTR—vaginal native tissue repair.

**Table 3:** Mean results of the female sexual function index (FSFI) questionnaire before and 6 months after surgery.

FSFI	Before	After 6 Months	*p*
All			
Mean ± SD	20.09 ± 8.09	23.82 ± 6.96	<0.001
VNTR			
Mean ± SD	20.52 ± 8.18	23.42 ± 7.64	<0.001
Synthetic material			
Mean ± SD	19.7 ± 8.04	24.2 ± 6.29	<0.001

SD—standard deviation, VNTR—vaginal native tissue repair.

**Table 4:** Spearman’s rank correlation coefficient (PISQ-12 and FSFI) depending on the type of surgery.

The Type of Surgery	T1	T2
TVM	0.60	0.75
VNTR	0.66	0.74

T1—before surgery; T2—6 months after surgery; VNTR—vaginal native tissue repair.

## Data Availability

The data is available with the author.
